# Hepatitis B Virus Pre-S Mutants as Biomarkers and Targets for the Development and Recurrence of Hepatocellular Carcinoma

**DOI:** 10.3390/v12090945

**Published:** 2020-08-26

**Authors:** Chiao-Fang Teng, Han-Chieh Wu, Ih-Jen Su, Long-Bin Jeng

**Affiliations:** 1 Graduate Institute of Biomedical Sciences, China Medical University, No.91, Hsueh-Shih Rd., Northern Dist., Taichung City 404, Taiwan; 2 Organ Transplantation Center, China Medical University Hospital, No.2, Yude Rd., North Dist., Taichung City 404, Taiwan; 3 Research Center for Cancer Biology, China Medical University, Taichung City 404, Taiwan; 4 National Institute of Infectious Diseases and Vaccinology, National Health Research Institutes, Zhunan 350, Taiwan; hanjie@nhri.org.tw; 5 Department of Biotechnology, Southern Taiwan University of Science and Technology, No.1, Nantai St., Yongkang Dist., Tainan City 710, Taiwan

**Keywords:** hepatitis B virus, hepatocellular carcinoma, biomarkers, targets, pre-S mutants

## Abstract

Chronic hepatitis B virus (HBV) infection is a major risk factor for the development of hepatocellular carcinoma (HCC), the leading cause of cancer-related death worldwide. Despite progress in the prevention and therapy of HCC, high incidence and recurrence rates of HCC remain big threats, resulting in poor patient survival. Effective biomarkers and targets of HCC are therefore urgently needed for better management and to improve patient outcomes. Pre-S mutants have been well demonstrated as HBV oncoproteins that play important roles in HCC development through activation of multiple oncogenic signal pathways in hepatocytes, in vitro and in vivo. The presence of pre-S mutants in patients with chronic HBV infection and HBV-related HCC has been associated with a significantly higher risk of HCC development and recurrence after curative surgical resection, respectively. In this review, we summarize the roles of pre-S mutants as biomarkers for predicting HBV-related HCC development and recurrence, and highlight the pre-S mutants-activated oncogenic signal pathways as potential targets for preventing HBV-related HCC development.

## 1. Introduction

Hepatocellular carcinoma (HCC) is among the most prevalent human cancers, and the leading causes of cancer-related death worldwide [1,2]. Despite remarkable progress made in developing diagnostics and therapeutics, high morbidity and mortality rates of HCC remain serious global public health problems [3,4]. Moreover, the high recurrence rate of HCC after curative surgical resection results in poor patient survival [5,6]. Therefore, the development of reliable biomarkers and targets for the development and recurrence of HCC are urgently needed to improve patient outcomes.

Chronic hepatitis B virus (HBV) infection is one of the major risk factors of HCC development, and contributes to over 50% of total HCC cases worldwide [7,8]. Several mechanisms have been proposed to explain HBV-induced hepatocarcinogenesis, such as the insertional mutagenesis and genomic instability caused by HBV DNA integration into host cell genomes, the chronic inflammation and regenerative hyperplasia initiated by immune responses to HBV infection, and the oncogenic functions elicited by HBV gene products [9,10]. There are four genes encoded by the HBV DNA genome, including the surface, core, polymerase, and X genes, which encode the surface antigens (HBsAg), core and e antigens (HBeAg), DNA polymerase, and X protein (HBx), respectively [11,12]. Because the HBV surface gene is composed of three gene segments (pre-S1, pre-S2, and S), each of which contains an in frame start codon, three different sizes of HBsAg are encoded, including the small, middle, and large HBsAg that are encoded by S, pre-S2 and pre-S1, in each gene segment, respectively [13].

Ground glass hepatocytes (GGHs) that harbor pre-S mutant proteins represent histologically preneoplastic lesions of HCC in patients with chronic HBV infection [14,15]. Two types of pre-S mutant proteins, the pre-S1 and pre-S2 mutants, are identified as the HBV large surface proteins that contain deletion mutations in the pre-S1 and pre-S2 gene segments, respectively [14,15]. Both types of pre-S mutants have been well demonstrated as HBV oncoproteins, that can activate multiple oncogenic signal pathways to promote growth advantage of hepatocytes, eventually leading to HCC development in vitro and in vivo [15,16,17,18]. Moreover, pre-S mutants have emerged as potential biomarkers and targets for HBV-related HCC.

In this review, we summarize the evidence that supports pre-S mutants as biomarkers for predicting and as targets for preventing the development and recurrence of HBV-related HCC (Table 1).

## 2. The Prevalence of Pre-S Mutants in Patients is Gradually Increased from Chronic Hepatitis B (CHB) to Liver Cirrhosis (LC) and Reaches a Peak in HCC

The natural course of chronic HBV infection can be divided into three sequential stages [28,29,30]. In the first stage, patients are HBeAg positive, and serum levels of HBV DNA are high. In the second stage, patients undergo HBeAg seroconversion and serum levels of HBV DNA decline. In the third stage, patients are HBeAg negative and serum levels of HBV DNA become low or undetectable. However, HBsAg persist in serum of nearly all patients through these three stages, and some patients in the third stage may progress to LC and ultimately HCC [28,29,30].

The incidence and pattern of pre-S mutants in patients with different stages of chronic HBV infection and liver diseases have been examined in several retrospective cohort studies (Table 2). Fan et al. screened pre-S deletion mutations in serum samples from 140 patients, at different replicative phases of chronic HBV infection by polymerase chain reaction (PCR)-based amplification of the entire pre-S gene (including the pre-S1 and pre-S2 gene segments), followed by TA cloning and DNA sequencing [31]. They analyzed 47 clones from 44 patients with high titers of HBV DNA (>10^8^ genomes/mL), 69 clones from 60 patients with intermediate titers of HBV DNA (10^8^ to 10^6^ genomes/mL), and 48 clones from 36 patients with low titers of HBV DNA (<10^6^ genomes /mL) [31]. The results showed that the prevalence of overall pre-S deletions (including the pre-S1 and/or pre-S2 deletions) was 6.4% at high replicative phase, 13% at intermediate replicative phase, and 37.5% at low replicative phase in serum [31]. Shen et al. performed similar analysis in another cohort of chronic HBV-infected patients, consistently showing that the prevalence of overall pre-S deletions in serum was as low as 7% at high replicative phase, but significantly increased to 13.9% and 37%, at intermediate and low replicative phases, respectively [32].

Moreover, Choi et al. conducted the PCR-based detection of pre-S deletions in serum samples from 250 patients with different HBV-related liver diseases, including 87 CHB patients, 91 LC patients, and 72 HCC patients [33]. They found that the prevalence of overall pre-S deletions in serum was 20.7% in CHB, 35.2% in LC, and 43.1% in HCC [33]. Li et al. performed similar analysis for the prevalence of overall pre-S deletions in serum samples from another cohort of patients, showing the same tendency, increasing significantly from 15.8% in CHB to 26.1% in LC and ultimately 34.6% in HCC [34]. Furthermore, their results indicated that the pre-S1 deletions were most frequently detected in LC, while the pre-S2 deletions were most frequently detected in HCC, accounting for 17.1% and 19.1%, respectively [34]. In another cohort study, Shen et al. showed that the prevalence of overall pre-S deletions in serum was even up to 60.3% in HBV-related HCC patients [32]. In addition, Jia et al. applied a next-generation sequencing (NGS)-based approach to detect pre-S deletions in serum samples from 45 CHB patients and nontumorous liver tissues from 94 HBV-related HCC patients [35]. The results also showed that the prevalence of overall pre-S deletions was significantly higher in HBV-related HCC than CHB (median percentage of the total nucleotides; 8.31% versus 2.75%) [35]. Zhao et al. conducted a capillary gel electrophoresis (CGE)-based approach, to analyze the prevalence of pre-S deletions in plasma samples from 157 HBV-related HCC and 160 non-HCC patients, consistently showing that pre-S deletions were more frequently detected in HCC than non-HCC (47.1% versus 28.1%; *p* value < 0.001) [36]. Furthermore, they found that the pre-S deletions of small size (≤99 base pairs), but not the pre-S deletions of large size (>99 base pairs), occurred more frequently in HCC than non-HCC (34.4% versus 15.0%; *p* value < 0.001) [36].

## 3. The Presence of Pre-S Mutants in Patients with Chronic HBV Infection is Associated with a Higher Risk of LC and HCC Development

Epidemiological studies have revealed that approximately 2% of CHB patients develop LC every year [37]. The annual incidence of HCC is about 1% in CHB patients, but increased to 3–10% in LC patients [38,39,40]. In addition, serum HBsAg persists in most CHB patients; HBsAg disappears from serum in only about 1% of CHB patients per year [41]. CHB patients with serum HBsAg have been shown to develop HCC with a 100-fold higher risk than those without [7].

The clinical correlation between the presence of pre-S mutants and the development of LC and HCC has been assessed in several prospective cohort studies. Chen et al. enrolled 141 HBeAg-negative patients without the development of LC and HCC, detected pre-S deletions from their serum samples by the PCR-based approach, and examined their clinical outcomes with a minimum follow-up period of 10 years [19]. The results showed that patients with pre-S deletions (either or both of pre-S1 and pre-S2 deletions) have a significantly 2.5- and 5-fold higher risk of developing LC (*p* value = 0.002) and HCC (*p* value < 0.0001) than those without, respectively [19]. Moreover, a multivariate analysis revealed that the presence of pre-S deletions were independent risk factors for LC (hazard ratio (HR), 2.95; 95% confidence interval (CI), 1.23–7.06; *p* value = 0.015) and HCC (HR, 11.25; 95% CI, 2.18–59.11; *p* value = 0.004) [19]. In another cohort study, Sinn et al. analyzed 195 CHB patients without HCC, among whom 79 had LC and 109 were HBeAg positive [20]. They observed a similar result, indicating that the 1-, 3-, and 5-year cumulative incidences of HCC were significantly higher in patients with pre-S deletions than those without (0, 7.4, and 26.5% versus 0.6, 4.1, and 5.7%; *p* value < 0.001) [20]. Multivariate analysis also showed that the presence of pre-S deletions was independently associated with an increased risk of HCC (HR, 3.04; 95% CI, 1.24–7.42; *p* value = 0.015) [20].

## 4. The Presence of Pre-S Mutants in Patients with HBV-Related HCC Predicts a Higher Risk of HCC Recurrence after Curative Surgical Resection

Although surgical resection is regarded as a potentially curative therapy for HCC patients, the recurrence rate of HCC is up to 80%, leading to poor overall survival rate of as low as 30% within 5 years after surgery [5,6]. Several prognostic biomarkers have been identified for the prediction of HCC recurrence, such as low levels of phosphatase and tensin homologue and high levels of p53 and proliferating cell nuclear antigen expression in tumor tissues [42,43]. In addition, high levels of serum HBsAg have been also proposed as independent risk factors for HCC recurrence after curative surgical resection [44].

Several studies have evaluated the clinical correlation between the presence of pre-S mutants and the recurrence of HCC after curative surgical resection. Tsai et al. enrolled 82 HBV-related HCC patients receiving curative surgical resection and detected the presence and pattern of GGHs in their nontumorous liver tissues by the immunohistochemistry (IHC) staining of HBV surface proteins [21]. Two types of GGHs (designated type I and II) have been identified to express different types of pre-S mutants with different intracellular distribution patterns; the type I GGHs express a globular or inclusion-like pattern of pre-S1 mutants, while the type II GGHs express a marginal or peripheral pattern of pre-S2 mutants [14,15]. Moreover, the type I GGHs display either sporadic or clustered growth patterns, whereas the type II GGHs are distributed consistently in clusters [14,15]. The authors applied a semiquantitative expression scoring system to quantify the pattern of GGHs, with scores from 0 to 4 corresponding to the percentage of positive immunostaining in 0%, <5%, 5% to 9%, 10% to 29%, and ≥30% of hepatocytes, respectively [21]. Their results showed that higher expression scores of the sporadic type I GGHs (score 1–4 versus 0; *p* value = 0.017), the clustered type I GGHs (score 4 versus 2–3 versus 0–1; *p* value = 0.004), and the type II GGHs (score 2–4 versus 0–1; *p* value < 0.001) were significantly associated with shorter local recurrence-free survival (LRFS); the type II GGHs (score 2–4 versus 0–1; *p* value = 0.005) were additionally associated with shorter overall survival (OS) [21]. Multivariate analysis revealed that the type II GGHs (score 2–4) were independently associated with decreased LRFS (HR, 4.048; 95% CI, 1.985–8.253; *p* value < 0.001) and OS (HR, 3.886; 95% CI, 1.605–9.412; *p* value = 0.003) [21]. Furthermore, in multivariate analysis, the type II GGHs (score 2–4) were identified as independent prognostic factors for the late (more than 1 year), rather than early (within 1 year), local tumor recurrence after surgery (HR, 3.407; 95% CI, 1.490–7.789; *p* value = 0.004) [21].

Moreover, Li-Shuai et al. detected pre-S deletions in serum samples from 113 HBV-related HCC patients receiving curative surgical resection by the PCR-based approach [22]. The results showed that patients with pre-S deletions displayed significantly higher HCC recurrence rates than those without (*p* value = 0.015) [22]. A multivariate analysis revealed that the presence of pre-S deletions were independent prognostic factors for HCC recurrence (HR, 1.781; 95% CI, 1.065–2.976; *p* value = 0.028) [22]. Furthermore, based on the pattern of pre-S deletions, they divided patients with pre-S deletions into several subtypes, and found that patients with the pre-S2 deletions only had significantly higher HCC recurrence rates than those with other patterns of pre-S deletions (HR, 2.211; 95% CI, 1.008–4.846; *p* value = 0.048) [22].

In addition, Yen et al. detected pre-S deletions in serum samples from 175 HBV-related HCC patients undergoing curative surgical resection by using a Pre-S Gene Chip-based approach, in which 30 independent pre-S clones in each serum sample were analyzed for semiquantitative detection of pre-S deletions [23]. The results showed that patients with HCC recurrence had significantly higher percentages of overall pre-S deletions (*p* value = 0.0189) and the pre-S2 deletions (*p* value = 0.0304), but not the pre-S1 deletions, than those without [23]. Moreover, higher percentages of the pre-S2 deletions were significantly associated with lower recurrence-free survival (RFS) and OS (≥5% versus <5% of the total clones; *p* value = 0.038 and *p* value = 0.047, respectively) [23]. A multivariate analysis revealed that the pre-S2 deletions (≥5%) were independently associated with an increased incidence of HCC recurrence after surgery (HR, 1.937; 95% CI, 1.401–2.678; *p* value < 0.001) [23].

Most recently, Teng et al. established a NGS-based platform for the detection of pre-S deletions in plasma samples [45,46] and applied it to analyze 75 HBV-related HCC patients receiving curative surgical resection [24]. Based on the distribution of deletions in either or both of the pre-S1 and pre-S2 gene segments of pre-S gene, they classified pre-S deletions into three types: the pre-S1, pre-S2, and pre-S1 + pre-S2 deletions [24]. According to the types and percentages of pre-S deletions, the patients were divided into several distinct groups and subgroups [24]. Their results showed that patients with either deletions spanning the pre-S2 gene segment (*p* value = 0.0283) or high percentages of pre-S2 plus pre-S1 + pre-S2 deletions (>24.995% versus ≤24.995% of the total pre-S gene DNA fragments; *p* value = 0.0089) or both factors (*p* value = 0.0067) had significantly poorer RFS than those without [24]. Furthermore, a multivariate analysis revealed that either the presence of deletions spanning the pre-S2 gene segment (HR, 2.114; 95% CI, 1.203–3.714; *p* value = 0.0092) or the percentage of pre-S2 plus pre-S1 + pre-S2 deletions (>24.995%; HR, 2.102; 95% CI, 1.148–3.850; *p* value = 0.0161), or a combination of both factors (HR, 2.336; 95% CI, 1.238–4.408; *p* value = 0.0088) was independently associated with a higher risk of HCC recurrence after surgery [24].

## 5. The Expression of Pre-S Mutants Predicts a Higher Risk of HCC Recurrence after Curative Surgical Resection, Even Under Pre-Surgical Anti-HBV Treatment

Because high levels of serum HBV DNA have been associated with HCC recurrence [47,48], anti-HBV treatment that inhibits HBV replication, hence reducing serum levels of HBV DNA, has been shown to decrease the recurrence rate of HCC in patients undergoing curative surgical resection [49,50]. For example, patients with nucleos(t)ide analogue (NA) treatment have been shown to have significantly lower recurrence rates and better survival following the curative surgical resection of HCC than those without [51]. However, there is still a high proportion of patients (up to 45%) suffering from HCC recurrence after surgery, despite receiving effective anti-HBV treatment [51].

The effect of anti-HBV treatment on the expression of pre-S mutants in liver tissues has been ascertained in a study by Tsai et al. [25]. They performed the IHC staining-based detection of GGHs in nontumorous liver tissues from 186 HBV-related HCC patients undergoing curative surgical resection, among whom 82 received NA treatment before surgery and 104 did not [25]. The results showed, that despite the reduction of serum HBV DNA levels, the expression scores of GGHs were not significantly decreased before and after 48 weeks of NA treatment (*p* value = 0.527 and *p* value = 0.077 for type I GGHs and type II GGHs, respectively) [25]. There was no significant difference in the expression scores of GGHs between the NA non-treatment and treatment groups of patients (*p* value = 0.594, *p* value = 0.811, and *p* value = 0.376 for total GGHs, type I GGHs, and type II GGHs, respectively) [25]. Moreover, no matter whether patients received pre-surgical NA treatment, higher expression scores of the type II GGHs (score 3–4 versus 0–2), but not the type I GGHs, were significantly associated with shorter LRFS (*p* value = 0.017 and *p* value = 0.036), especially for LRFS more than 1 year after surgery (*p* value = 0.017 and *p* value = 0.036 for the NA treatment and non-treatment groups of patients, respectively) [25]. Multivariate analysis revealed that the type II GGHs (score 3–4) were independent prognostic factors for local tumor recurrence after surgery, in both NA non-treatment (HR, 2.455; 95% CI, 1.162–5.190; *p* value = 0.019) and treatment (HR, 4.488; 95% CI, 1.992–10.113; *p* value = 0.001) groups of patients [25].

## 6. The Oncogenic Signal Pathways Activated by Pre-S Mutants Represent Potential Targets for Chemoprevention of HBV-Related HCC

**Figure 1:**
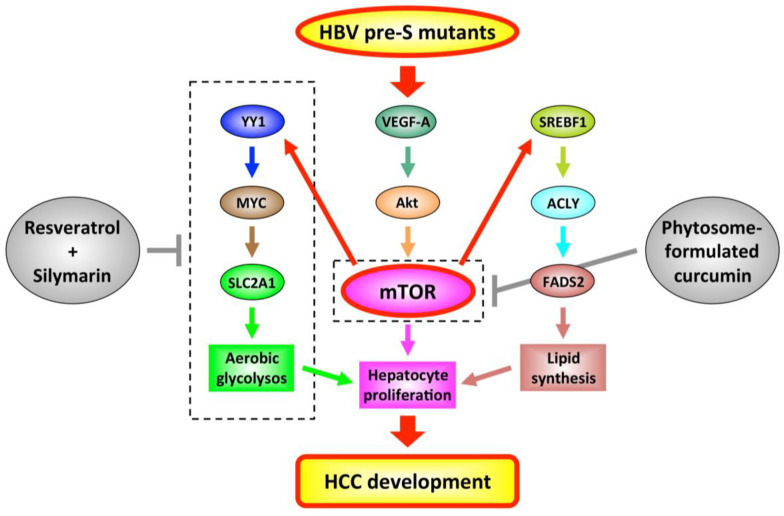
Schematic representation of HBV pre-S mutants-activated mTOR signal pathways as potential targets for HCC chemoprevention. In chronic HBV infection, pre-S mutants can upregulate the expression of vascular endothelial growth factor-A (VEGF-A) in GHHs. Through binding to its cognate receptor, the upregulated VEGF-A functions in either autocrine or paracrine manners to activate mTOR via mediation of Akt activation. The activated mTOR can promote hepatocyte proliferation directly or indirectly, by initiating two metabolic pathways, one involving Yin Yang 1 (YY1)/MYC/solute carrier family 2 (facilitated glucose transporter) member 1 (SLC2A1) to induce aerobic glycolysis, and another involving sterol regulatory element-binding factor 1 (SREBF1)/adenosine triphosphate citrate lyase (ACLY)/fatty acid desaturase 2 (FADS2) to stimulate lipid synthesis. Combined effects of hepatocyte proliferation, aerobic glycolysis, and lipid synthesis will potentially result in HCC development. By means of blocking the pre-S mutants-activated mTOR signal pathways, treatment of either the resveratrol combined with silymarin or the phytosome-formulated curcumin exhibits potential efficacy in preventing HCC development. Abbreviations: HBV, hepatitis B virus; VEGF-A, vascular endothelial growth factor-A; mTOR, mammalian target of rapamycin; YY1, Yin Yang 1; SLC2A1, solute carrier family 2 (facilitated glucose transporter) member 1; SREBF1, sterol regulatory element-binding factor 1; ACLY, adenosine triphosphate citrate lyase; FADS2, fatty acid desaturase 2; HCC, hepatocellular carcinoma.

It has been well demonstrated that pre-S mutants play important roles in the development of HBV-related HCC through the activation of multiple oncogenic signal pathways to promote cell cycle progression, proliferation, survival, and metabolism in hepatocytes [15,16,17]. For example, pre-S mutants have been shown to activate mammalian target of rapamycin (mTOR) pathways to induce hepatocyte proliferation directly or indirectly, through the stimulation of aerobic glycolysis and lipid synthesis (Figure 1) [26,52,53]. The expression of pre-S2 mutants in transgenic mice results in liver inflammation and fibrosis, hepatomegaly, and HCC formation, along with the consistent activation of mTOR signal pathways throughout the liver tumorigenesis [18]. In addition, some GGHs have been observed to co-express pre-S2 mutant and another HBV oncoprotein, HBx [54]. The transgenic mice expressing both pre-S2 mutant and HBx proteins exhibit the stronger activation of mTOR signal pathways and higher incidence (100%) of HCC than those expressing pre-S2 mutants alone [54].

Considering the advantages, such as low toxicity, easy availability, and multitarget properties, natural products have emerged as promising chemopreventive and therapeutic agents for cancer [55]. Two studies by Teng et al. have used the transgenic mice expressing both pre-S2 mutant and HBx proteins as a model of HBV-related HCC, to validate the efficacy of natural products in suppressing pre-S mutants-activated oncogenic signal pathways and preventing HCC development [26,27]. The first study showed that combination treatment of the resveratrol and silymarin [56,57], two botanical compounds derived from the grape skin and milk thistle, respectively, could significantly suppress HCC formation through the inhibition of the mTOR-dependent glycolysis pathways (Figure 1) [26]. The second study demonstrated that the treatment of the phytosome-formulated curcumin [58], a botanical compound derived from the turmeric, could significantly improve liver histopathology, decrease lipid accumulation and leukocyte infiltration in liver, and suppress HCC formation through the activation of anti-inflammatory peroxisome proliferator-activated receptor γ (PPARγ), suppression of pro-inflammatory nuclear factor-κB (NF-κB), and inhibition of oncogenic mTOR activation (Figure 1) [27].

## 7. Conclusions

Despite substantial progress made in the prevention and therapy, valuable biomarkers and targets for HCC development and recurrence remain necessary to improve patient outcomes. This review highlights that the presence, types, and levels of HBV pre-S mutants in serum, plasma, and nontumorous liver tissues represent independent biomarkers for predicting HCC development and recurrence after curative surgical resection, even under effective anti-HBV treatment. Targeting at the pre-S mutants-activated oncogenic signal pathways may be a promising strategy for HBV-related HCC chemoprevention.

## Figures and Tables

**Table 1:** The roles of pre-S mutants as biomarkers and targets for hepatitis B virus (HBV)-related hepatocellular carcinoma (HCC) development and recurrence.

I. Biomarkers			
Ref.	Sample Type	Detection Approach	Summary of the Evidence
Chen et al. [19]	Serum	PCR	Higher risk of HCC development in patients with pre-S deletions (either or both of pre-S1 and pre-S2 deletions)
Sinn et al. [20]	Serum	PCR	Higher risk of HCC development in patients with pre-S deletions (either or both of pre-S1 and pre-S2 deletions)
Tsai et al. [21]	Nontumorous liver tissues	IHC staining	Higher risk of HCC recurrence after curative surgical resection in patients with the type II GGHs (pre-S2 deletions; score 2–4)
Li-Shuai et al. [22]	Serum	PCR	Higher risk of HCC recurrence after curative surgical resection in patients with pre-S deletions, especially in patients with the pre-S2 deletions only
Yen et al. [23]	Serum	Pre-S Gene Chip	Higher risk of HCC recurrence after curative surgical resection in patients with the pre-S2 deletions (≥5%)
Teng et al. [24]	Plasma	NGS	Higher risk of HCC recurrence after curative surgical resection in patients with either deletions spanning the pre-S2 gene segment or high percentage of pre-S2 plus pre-S1 + pre-S2 deletions (>24.995%) or both factors
Tsai et al. [25]	Nontumorous liver tissues	IHC staining	Higher risk of HCC recurrence after curative surgical resection in patients with the type II GGHs (pre-S2 deletions; score 3–4) even under pre-surgical anti-HBV treatment
**II. Targets**			
**Ref.**	**Mouse Model**	**Treatment**	**Summary of the Evidence**
Teng et al. [26]	Transgenic mice expressing both pre-S2 mutant and HBx proteins	Combination of resveratrol and silymarin	Suppression of HCC formation through inhibition of mTOR-dependent glycolysis pathways
Teng et al. [27]	Transgenic mice expressing both pre-S2 mutant and HBx proteins	Phytosome-formulated curcumin	Suppression of HCC formation through activation of PPARγ and inhibition of NF-κaB and mTOR activation

Abbreviations: PCR, polymerase chain reaction; IHC, immunohistochemistry; NGS, next-generation sequencing; HCC, hepatocellular carcinoma; GGHs, ground glass hepatocytes; HBx, hepatitis B virus X; mTOR, mammalian target of rapamycin; PPARγ, peroxisome proliferator-activated receptor γ; NF-κB, nuclear factor-κB.

**Table 2:** The prevalence of pre-S mutants in patients with different stages of chronic HBV infection and liver diseases.

Ref.	Sample Type	Detection Approach	Summary of the Evidence
Fan et al. [31]	Serum from patients at low, intermediate, and high HBV replicative phases	PCR	Higher prevalence of overall pre-S deletions (including the pre-S1 and/or pre-S2 deletions) at low HBV replicative phase
Shen et al. [32]	Serum from patients at low, intermediate, and high HBV replicative phases	PCR	Higher prevalence of overall pre-S deletions (including the pre-S1 and/or pre-S2 deletions) at low HBV replicative phase
Shen et al. [32]	Serum from CHB and HCC patients	PCR	Higher prevalence of overall pre-S deletions (including the pre-S1 and/or pre-S2 deletions) in HCC
Choi et al. [33]	Serum from CHB, LC, and HCC patients	PCR	Higher prevalence of overall pre-S deletions (including the pre-S1 and/or pre-S2 deletions) in HCC
Li et al. [34]	Serum from CHB, LC, and HCC patients	PCR	Higher prevalence of overall pre-S deletions (including the pre-S1 and/or pre-S2 deletions) in HCC; predominance of pre-S1 and pre-S2 deletions in LC and HCC, respectively
Jia et al. [35]	Serum from CHB and nontumorous liver tissues from HCC patients	NGS	Higher prevalence of overall pre-S deletions (including the pre-S1 and/or pre-S2 deletions) in HCC
Zhao et al. [36]	Plasma	CGE	Higher prevalence of overall pre-S deletions (including the pre-S1 and/or pre-S2 deletions) in HCC; predominance of the pre-S deletions of small size (≤99 base pairs) in HCC

Abbreviations: HBV, hepatitis B virus; CHB, chronic hepatitis B; LC, liver cirrhosis; HCC, hepatocellular carcinoma; PCR, polymerase chain reaction; NGS, next-generation sequencing; CGE, capillary gel electrophoresis.
